# Experiences and Acceptability of a Weight Loss Intervention for Diabetes (Diabetes Remission Clinical Trial—DiRECT) in Aotearoa New Zealand: A Qualitative Study within a Pilot Randomised Controlled Trial

**DOI:** 10.3390/nu16121853

**Published:** 2024-06-13

**Authors:** Kate Campbell, Meredith Peddie, Natalie Ashton, Kim Ma’ia’i, Takiwai Russell-Camp, Jim Mann, Justine Camp, Andrew N. Reynolds

**Affiliations:** 1 Department of Medicine, University of Otago, Dunedin 9054, Aotearoa, New Zealand; kate.campbell@postgrad.otago.ac.nz (K.C.); takiwai.russell-camp@otago.ac.nz (T.R.-C.); jim.mann@otago.ac.nz (J.M.); justine.camp@otago.ac.nz (J.C.); 2 Department of Human Nutrition, University of Otago, Dunedin 9054, Aotearoa, New Zealand; meredith.peddie@otago.ac.nz; 3 Edgar Diabetes and Obesity Research Centre, Dunedin 9054, Aotearoa, New Zealand; 4 Te Kāika Health, Caversham 9012, Aotearoa, New Zealand; natalie.ashton@wellsouth.org.nz (N.A.); kimmaiai33@gmail.com (K.M.)

**Keywords:** type 2 diabetes, weight loss, total diet replacement, qualitative analysis

## Abstract

The Diabetes Remission Clinical Trial (DiRECT) demonstrated that substantial weight loss and remission from type 2 diabetes can be achieved with low-energy total diet replacement and behavioural support. However, the acceptability of the DiRECT intervention in diverse populations with strong cultural emphases on food and shared eating remains unclear. We conducted a qualitative study nested within a pilot randomised controlled trial of DiRECT in one Māori (the Indigenous people of New Zealand) primary care provider in Aotearoa New Zealand. Participants with type 2 diabetes or prediabetes, obesity, and a desire to lose weight were randomised to either dietitian-supported usual care or the dietitian-supported DiRECT intervention for twelve months. The DiRECT intervention included three months of total diet replacement, then food reintroduction and supported weight loss maintenance. At three and twelve months, semi-structured interviews explored the acceptability of DiRECT and participants’ experiences of each intervention. Interview transcripts from 25 participants (aged 48 ± 10 years, 76% female, 78% Māori or Pacific) at three months and 15 participants at twelve months were analysed. Participants viewed their pre-enrolment selves as unhealthy people with poor eating habits and desired professional weight loss support. For DiRECT participants, the total diet replacement phase was challenging but well-received, due to rapid improvements in weight and health. Food reintroduction and weight loss maintenance each presented unique challenges requiring effective strategies and adaptability. All participants considered individualised and empathetic dietetic support crucial to success. Sociocultural factors influencing success were experienced in both interventions: family and social networks provided support and motivation; however, eating-related norms were identified as challenges. The DiRECT intervention was considered an acceptable approach to weight loss in participants with type 2 diabetes or prediabetes with strong cultural emphases on food and shared eating. Our findings highlight the importance of individualised and culturally relevant behavioural support for effective weight loss and weight loss maintenance.

## 1. Introduction

Type 2 diabetes has been termed a “defining disease of the 21st century” requiring urgent global action [1] (p. 2087). While previously regarded as an irreversible and inevitably progressive condition, it is now understood that remission from type 2 diabetes is possible [2,3,4] and should be a primary therapeutic target at the time of diagnosis. The original Diabetes Remission Clinical Trial (DiRECT) [5] and several subsequent studies [6,7,8,9] demonstrated that substantial weight loss and remission from type 2 diabetes could be achieved via lifestyle interventions. The DiRECT intervention consists of a period of total diet replacement (typically three months) followed by food reintroduction and a longer period of supported weight loss maintenance. In the United Kingdom, evidence from the original trial [5] prompted public funding of DiRECT as a national “diabetes remission service” [10] (p. 1) available to adults with type 2 diabetes, obesity, and a desire to lose weight [11].

The success of any dietary intervention is invariably linked to its acceptability or social validity [12,13]. Previous qualitative research has found DiRECT to be acceptable among people with type 2 diabetes [14,15,16] in studies of predominantly European populations. However, food and mealtimes serve an important symbolic purpose in many cultures globally, which may influence the acceptability of DiRECT elsewhere. Eating practices such as shared mealtimes reflect and reaffirm social values, beliefs, and identities [17,18,19]. Eating together has remained integral to life in many cultures, despite its diminished importance in western societies [20].

To date, the impact and acceptability of DiRECT in diverse populations with strong cultural emphases on food and shared eating have not been considered. Aotearoa New Zealand is a nation of diverse ethnic, social, and cultural heritage [21] with equally diverse perspectives surrounding health and wellbeing. For example, Māori (the Indigenous people of Aotearoa New Zealand) and Pacific Island ethnicities comprise one-quarter of New Zealand’s population [22]. Whereas a western worldview tends to define ‘health’ in largely physical terms, Māori and Pacific cultures share a holistic perspective where mental, emotional, social, spiritual, and physical health are inextricably connected [23,24,25]. Through this lens, “food as wellbeing” [19] (p. 5) is a central construct, and food nourishes every aspect of health [26,27,28].

We have conducted a qualitative study to consider the acceptability of the DiRECT intervention in a population group with strong cultural emphases on food and shared eating. This was nested within a pilot randomised controlled trial comparing DiRECT with dietitian-supported usual care, enabling us to comment on the participants’ experiences of both twelve-month interventions. 

## 2. Materials and Methods

### 2.1. Study Design

Qualitative interviews to capture participants’ perspectives and experiences were undertaken within a pilot randomised controlled trial at three and twelve months. The trial was conducted at a single kaupapa Māori primary care provider (a health service guided by Māori principles) at two locations between October 2021 and December 2023. Participants were randomised to either three months of dietitian-supported low-energy total diet replacement followed by nine months of weight loss maintenance (DiRECT), or twelve months of dietitian-supported usual care (UC).

Ethical approval for the trial was obtained (SHDEC 2022 EXP 11570), and the trial was prospectively registered (ACTRN12622000151730). All participants provided informed written consent prior to enrolment and were free to withdraw at any time with no disadvantage. This study was participant-driven, conducted in response to patients with type 2 diabetes wanting weight loss support at the primary care provider. All aspects of the study design, analysis, and reporting were informed by a multicultural research team, including those with expertise in kaupapa Māori research methodologies. Our reporting is guided by the consolidated criteria for reporting qualitative research (COREQ) [29] (see Supplementary Material: completed COREQ checklist).

### 2.2. Participants

Eligible participants were aged 20–65 years and had type 2 diabetes or prediabetes (glycated haemoglobin/HbA1c ≥ 39 mmol/mol), obesity (body mass index ≥ 30 kg/m^2^), and a self-reported desire to lose weight. Those with current insulin or anti-obesity medication use, heart failure, myocardial infarction in the previous six months, a diagnosed eating disorder, cancer, or a current pregnancy were not eligible.

### 2.3. Interventions

The dietitian-supported usual care (UC) arm consisted of 21 scheduled consults over twelve months with the primary care provider’s in-house dietitian. Nutrition education and advice were tailored to participants’ needs and preferences.Frequently-addressed topics included macronutrients, portion sizes, practical weight loss strategies, and the management of relevant health conditions. A gradual increase in physical activity to align with current guidelines [30] was encouraged, but no specific diet or exercise regimens were prescribed.

The DiRECT intervention also consisted of 21 scheduled consults with the same dietitian over twelve months. The DiRECT intervention was delivered in three phases: 12 weeks of total diet replacement with low-energy formula products (~3600 kJ per day), 4–6 weeks of food reintroduction, then supported weight loss maintenance for the remainder of the twelve-month intervention. All diet replacement products (Cambridge Weight Plan Ltd., Corby, Northants, UK) were provided free-of-charge in various formats (e.g., shakes, bars, and meals) and flavours. If required, participants were advised to increase water intake and/or add green vegetables (up to two cups per day) to manage satiety and avoid constipation during the total diet replacement phase. Dietetic consults during the total diet replacement phase addressed adherence, adverse effects, and the provision of additional products. Following food reintroduction, these consults mirrored those offered in usual care. All oral glucose-lowering agents and antihypertensive medications were withdrawn upon intervention commencement to minimise the risk of hypoglycaemia and hypotension, respectively. These medications were reintroduced if deemed necessary by a general practitioner (GP) or pharmacist. Participants were encouraged to continue their usual physical activities. 

All participants continued to receive routine diabetes care [31] and free access to an onsite gym during the twelve-month intervention. All enrolled participants were invited to take part in a one-on-one interview regarding their experiences in this study upon review at three months and twelve months. All participants who agreed were interviewed. 

### 2.4. Interview Procedure

The interviews followed a semi-structured interview guide (see Supplementary Material: semi-structured interview guides at 3 and 12 months). The interview guides were designed with input from experts in kaupapa Māori, dietetics, endocrinology, and primary care. These were amended according to feedback from one qualitative researcher experienced in questionnaire development but were not pilot tested. The questions were developed to prompt discussion on topics including general intervention experiences, intervention impacts, translations into behaviour change, and future expectations. Some specific questions were asked only of the DiRECT participants (e.g., experiences of food reintroduction). The interviewer was free to ask additional questions or prompt for more information where relevant, focusing on issues pertinent to the participant and facilitating deep exploration of the “insider perspective” [32] (p. 4).

The interviews were conducted by research staff not involved in the intervention delivery, almost entirely via telephone. All but one interview was conducted by a young, female, non-Māori, non-Pacific researcher who was a PhD student with experience conducting nutrition-related qualitative interviews. The participants had not previously met the interviewer and were informed that she was a researcher interested in lifestyle interventions for people with type 2 diabetes and prediabetes. Two participants elected to have a support person (spouse or friend) present to assist in translating, comprehending, and responding to questions. All the interviews were audio-recorded. Field notes were not made. The interviews lasted an average of 25 min at three months and 29 min at twelve months. 

### 2.5. Data Analysis

The interviews were automatically transcribed using Zoom software (version 5.8.3, Zoom Video Communications Inc., San Jose, CA, USA). Ttranscripts were checked against the audio recordings to ensure the responses were captured verbatim. The transcripts were emailed to participants for review prior to our analysis, but participants were not invited to comment on the findings. One participant withdrew their entire three-month transcript, but no other alterations were made.

The transcripts were anonymised and uploaded to the NVivo software (release 1.7.1, Lumivero LLC, Denver, CO, USA) for the analysis using a recursive six-phase process of reflexive thematic analysis [33,34,35]. (1) Familiarisation: this involved the reading and re-reading of the transcripts and the identification of potential themes. At this stage, potential themes largely mirrored the question topics addressed in the interviews (e.g., the challenges of total diet replacement), which would be refined in subsequent phases. (2) Coding: this involved applying descriptive and in-vivo labels to potentially relevant excerpts based on their meaningfulness [34,36]. The coding was predominantly inductive and data-driven; however, a degree of deductive coding ensured that ideas pertinent to the research aims (e.g., both positive and negative experiences) were captured. The text surrounding core terms was coded to retain the context of participants’ responses. Coding was neither exclusively semantic nor latent; for example, the excerpt, “I knew what needed to be done, it’s just the motivation and self-discipline”, (P01, DiRECT, 3 months, not published) could be coded for its explicit meaning using a semantic code (e.g., ‘self-motivation facilitates success’), its more implicit connotations using a latent code (e.g., ‘motivational support is just as important as nutrition education’), or both. This approach encouraged reflexivity in that the coder had to continually reflect on their own assumptions and interpretations to code responses in terms of direct (what the participant said) and indirect meanings. (3) Generating initial themes: this involved grouping related codes to develop candidate themes specific to DiRECT and across both interventions. (4) Developing and reviewing themes: this involved evaluating candidate themes against coded data and the entire dataset to ensure “patterns of shared meaning” [33] (p. 593) were clearly captured. (5) Refining, defining, and naming themes: this involved producing a structurally coherent set of themes (and sub-themes) with distinct meanings. As in Phase 1, the candidate themes generated in phase (3) predominantly reflected (i) the topics addressed in the interviews; and (ii) time relative to enrolment in this study (i.e., before, during, or beyond participation). It was decided that these represented domain summaries (i.e., shared topics) rather than meaningful patterns, and the initial thematic structure was set aside. Codes and themes were revised and re-organised with input from the authors. Broad themes were broken into smaller, more specific themes; overlapping themes were collapsed; ideas were shifted between themes; and superfluous content was discarded. For example, the broad candidate theme ‘Experiences and expectations before the study’ was restructured to encompass two final themes: ‘Unhealthy Habits and Unhelpful Advice’ and ‘An Opportunity for Change’. (6) Write-up: this involved “weaving together the analytic narrative” [35] and selecting quotes to illustrate each theme.

Our analysis is underpinned by constructionist theory, which states that reality is subjective and our perspectives are shaped by personal experiences within unique social contexts [37]. Participants’ responses therefore reflect their own realities rather than objective truths about the nature of either intervention. As such, ‘data saturation’ was not actively sought nor measured [38,39]. A constructionist approach also requires reflexivity: the recognition of a researcher’s background, biases, and assumptions, and their potential influence on the research process [33,40]. Multiple coders were not used as reflexive thematic analysis prioritises “reflective and thoughtful engagement” with the data and its analysis [33] (p. 594), as opposed to seeking inter-researcher consensus. However, eight coded transcripts were reviewed by another researcher to provide an alternative perspective and to strengthen the coding framework. Two researchers with expertise in kaupapa Māori and one in Pacific culture offered feedback on aspects of the analysis relevant to culture and cultural identity.

### 2.6. Sample Size

This trial was primarily conducted to assess the acceptability of the DiRECT intervention in Aotearoa New Zealand through a qualitative analysis. There are no precise guidelines for the number of interviews required in qualitative health research [32,41]. While we did not actively seek data saturation, the qualitative sample size determination is frequently based on this concept [38,39]. A sample of 9–17 participants is typically required to achieve saturation, including studies conducted in this participant population [42]. We recruited a sample of 40 participants into the randomised controlled trial. We expected this larger sample size to sufficiently capture the widest possible range of perspectives and lived experiences, to identify patterns across the dataset, and to address our research questions [43,44,45]. All participants who remained enrolled in this study were invited via telephone to an interview at each timepoint.

## 3. Results

**Figure 1:**
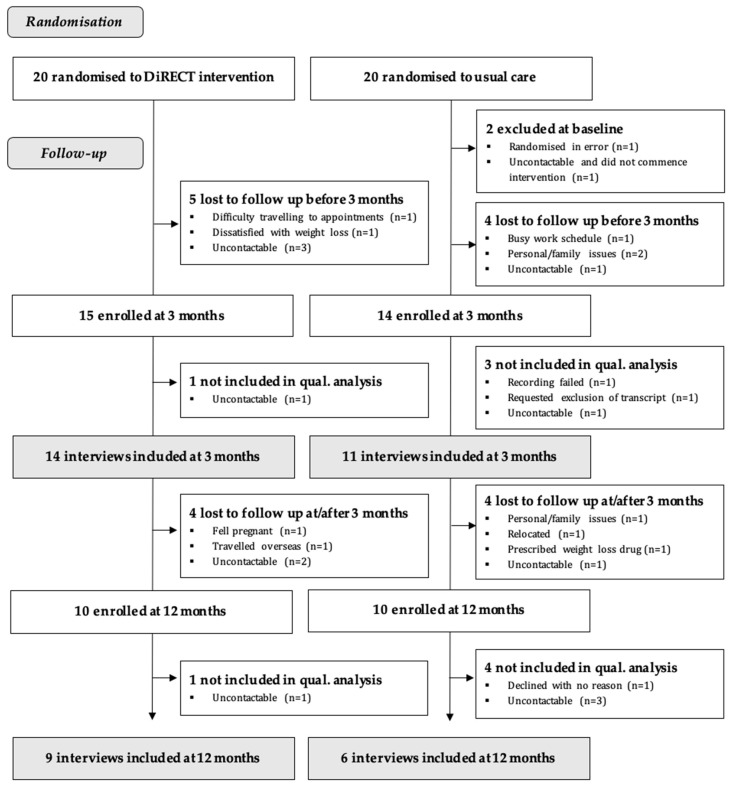
Flow of participants through the pilot randomised controlled trial and qualitative study.

Forty participants were randomised at baseline (*n* = 20 to DiRECT; *n* = 19 to dietitian-supported usual care; *n* = 1 in error), as shown in Figure 1. The proportion of the participants retained and interviewed was similar between interventions at each timepoint. Fourteen DiRECT participants (70%) were interviewed at three months and ten (50%) at twelve months. Thirteen usual care participants (68%) were interviewed at three months (two transcripts were not analysed, because the recording failed or it was excluded at the request of the participant) and ten (52%) at twelve months. The participants included in this qualitative analysis were aged 48 ± 10 years and were predominantly female (76%) and of Māori (54%) or Pacific (24%) ethnicity. To protect participants’ identities, limited individual data are presented in Table 1.

**Figure 2:**
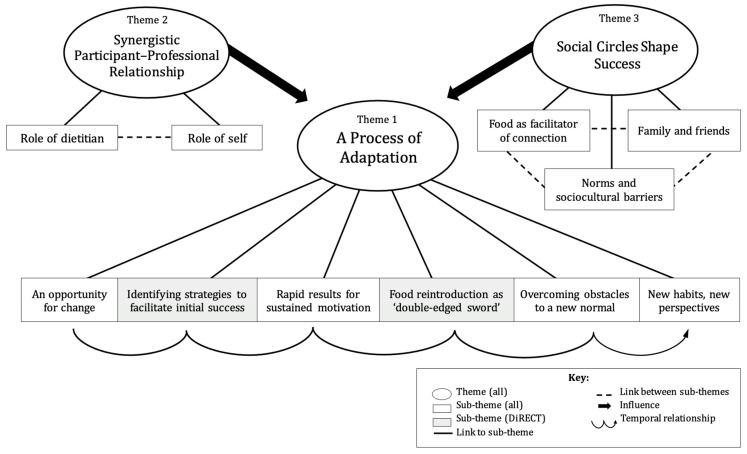
Final thematic map illustrating relationships between themes and sub-themes.

As illustrated in Figure 2, we developed three overarching themes, each presented as several sub-themes. The central theme (‘A Process of Adaptation’) expresses a temporal transformation of behaviour, experience, and perspective across the twelve-month duration of each intervention. In contrast, the remaining two themes (‘A Synergistic Relationship between Participant and Professional’ and ‘Social Circles Shape Success’) illustrate the salient role of interpersonal influences that remained relevant to participants’ experiences at every timepoint. During our analysis, it became evident that the experiences of each intervention were uniquely personal, despite many similarities. Our analysis therefore reflects common perspectives alongside conflicting ones and reflects on several similarities and differences between participants’ experiences of DiRECT and dietitian-supported usual care.

### 3.1. A Process of Adaptation

Each phase of the DiRECT intervention (total diet replacement, food reintroduction, and weight loss maintenance) came with its own advantages and difficulties. However, the dietitian-supported usual care participants also found weight loss challenging. Obstacles would emerge and resurface throughout each intervention, requiring adaptability, resilience, and professional guidance. As such, participants underwent a gradual process of adaptation. This is illustrated via six chronological sub-themes, beginning with participants’ (often negative) experiences and expectations prior to enrolment; examining the challenges, benefits, and strategies identified at each intervention phase; and concluding with the physical, psychological, and behavioural transformations apparent at twelve months.

#### 3.1.1. An Opportunity for Change

Across both interventions, participants reflected on their pre-enrolment selves as unhealthy people with poor eating habits and an inability to implement behaviour change. A sense of desperation was often implied: participants wanted to improve their health, weight, and quality of life but felt powerless in doing so. Therefore, participation in this study represented an opportunity to create meaningful change.

I know I was so overweight. I could feel it. I could feel my health declining … I knew I had to do something.

(P37, DiRECT, 3 months)

Most participants reported a long and futile history of attempts at losing weight: usually ‘quick fixes’ resulting in some weight loss, followed by rapid regain.

I’ve battled with my weight for years and years … I’ve been to Weight Watchers over the years and tried fad diets and stuff like that …

(P33, UC, 3 months)

Everything I’ve tried has been short term and then I end up putting more weight on.

(P28, DiRECT, 12 months)

Participants identified an unmet need for individualised weight management advice, though the type and level of guidance required differed. Some desired motivational support and a structured approach, while others wanted to gain new skills and knowledge.

I know what I need to do, what I should do, but for me just motivation is probably an issue.

(P08, DiRECT, 3 months)

[I needed] somebody to guide me somewhere, start me somewhere … I didn’t know how to start or where to start.

(P07, DiRECT, 3 months)

I needed that expert advice.

(P43, DiRECT, 3 months)

#### 3.1.2. Seeking Strategies to Facilitate Initial Success

Participants in the DiRECT intervention had to adapt to the unfamiliarity of the total diet replacement phase. This was physically and mentally demanding but became less so over time and was often perceived as easier than anticipated.

Those first three days were incredibly challenging … I felt like I had no energy whatsoever, but after that … the more I got into it the more I felt I could stick to it.

(P28, DiRECT, 3 months)

It wasn’t as hard as I expected.

(P25, DiRECT, 3 months)

Once you got used to it, it was pretty easy.

(P08, DiRECT, 12 months)

While some experienced physical hunger and a lack of energy, the psychological ‘test’ of not consuming ‘real food’ and adjusting to small portion sizes was often a much greater challenge.

I reckon it’s the mind game more than anything … to have, like, a shake as a meal.

(P20, DiRECT, 3 months)

Total diet replacement offered a balance between autonomy and control, promoting adherence. The products were nutritionally complete, required minimal preparation, and the structured plan removed the mental burden of healthy decision making. The variety of products and flavours on offer satisfied desires for ‘real food’ and reduced the monotony of the diet. Although some participants reported mild digestive issues or disliking the taste of the diet replacement products initially, these issues were typically temporary. For one participant, side effects resulted in early food reintroduction.

I like things that are prescriptive. You’ve got a plan.

(P25, DiRECT, 3 months)

All I had to do was pick the next one.

(P35, DiRECT, 3 months)

It was really nice to have the variety. I think it made a huge difference.

(P20, DiRECT, 3 months)

Strategies to quell hunger and satisfy cravings included drinking plenty of water and adding low-energy flavourings (e.g., chilli). Meal replacement bars could be broken into pieces for snacking across the day, instead of being consumed all at once. Using smaller dinnerware and seeking distractions from food also helped to ease the psychological challenges of total diet replacement.

I would cut a bar into three, so I would have a piece for morning tea, a piece for afternoon tea, and a piece for supper, and that worked really well.

(P20, DiRECT, 3 months)

I tried to sort of trick myself in terms of [eating smaller portions] by using either a small plate or a small bowl, and a teaspoon.

(P25, DiRECT, 3 months)

A sense of routine and structure further supported adherence during the total diet replacement phase, whereas unexpected changes, irregular schedules, and stressful life events (e.g., travelling for work, moving house, or dealing with illness) were considered disruptive.

Being busy helped. On my days off I actually found it harder.

(P37, DiRECT, 3 months)

I had to go to Christchurch for a week to work, I had to go to Auckland for a week to work, and those were the most challenging times, because I was away from my normal patterns and out of routine.

(P28, DiRECT, 3 months)

Adaptability and resilience were essential when participants had to deviate from the prescribed regimen. While some participants moved on from these situations readily, others saw any deviation as a barrier to progress.

If I was really really struggling, I’d say, bugger it, I’m gonna have something to eat, and then get back on the wave.

(P43, DiRECT, 3 months)

There were some days that I needed to eat differently and then it was harder getting back onto it once I’d come off it.

(P28, DiRECT, 3 months)

#### 3.1.3. Rapid Results for Sustained Motivation

Early and rapid weight loss was an important source of motivation for the DiRECT participants, and comments from others provided additional encouragement.

I got more motivated as time went on, ‘cause you’re starting to see small changes, and then people start commenting on small changes.

(P08, DiRECT, 3 months)

You see results quickly, and those results motivate you to keep going.

(P25, DiRECT, 3 months)

During the first three months, participants saw benefits beyond weight loss alone, including improvements in energy levels, sleep quality, and their general sense of wellbeing, providing further motivation.

Certainly seeing the results in terms of blood sugars and … sleeping better, having more energy, just generally feeling better also, as a result of what I’ve been doing, has been keeping me motivated.

(P25, DiRECT, 3 months)

The usual care participants also identified small yet positive shifts in their physical and mental wellbeing, but slow weight loss tended to stifle motivation.

I’m just trying to muddle along, but don’t feel like I’m making progress really.

(P19, UC, 3 months)

I’ve almost thought about going back to my doctor and asking if I could go back on some pills I was on a few years back.

(P24, UC, 3 months)

In contrast, a key advantage of the DiRECT intervention was the ‘kickstart’ it provided, motivating ongoing change and improvement among participants.

What’s made the difference now is, one, the kickstart of those first weeks on using the supplements, and now, the follow ups, the regular follow ups to make sure I’m on track.

(P25, DiRECT, 3 months)

#### 3.1.4. Food Reintroduction: A ‘Double-Edged Sword’

For some DiRECT participants, food reintroduction was viewed as an inevitable and welcome next step. Others felt that returning to ‘real food’ interrupted the routine and regimen they had become accustomed to.

Going back to real food didn’t really phase me a lot, and like I said, [TDR] gave me a good reset.

(P08, DiRECT, 12 months)

I’m just trying to prevent myself from going back to those bad habits now that I’m slowly going back onto real food. I’m finding it hard.

(P37, DiRECT, 3 months)

While food reintroduction came with a variety of advantages, it also posed several perceived risks to progress.

Double-edged sword.

(P01, DiRECT, 3 months)

Participants were excited about enjoying ‘real food’ again and looked forward to participating in family mealtimes and social activities without hesitation.

I think it’s just the psychological side of eating normal food … there’s nothing like the feeling of chewing food.

(P08, DiRECT, 3 months)

I feel as though now I can actually interact more socially.

(P25, DiRECT, 3 months)

However, concerns were raised about the time, money, and effort required to plan, purchase, and prepare healthy food, each of which had been alleviated during total diet replacement.

Now I’ve gotta think, and now I’ve gotta meal plan, and now it’s just something else that I’ve gotta do.

(P28, DiRECT, 12 months)

The absence of a regimented diet plan and the sense of control it had facilitated during total diet replacement was considered the greatest disadvantage of food reintroduction. Some participants were concerned they may suffer a lapse in motivation, revert to previous eating habits, and rapidly regain weight.

When you come off [TDR] that kinda restriction had disappeared, then it becomes a bit of a battle with portion sizing.

(P01, DiRECT, 12 months)

That’s always in the back of my mind that because it’s not so controlled, so prescriptive, that I could fall off the wagon.

(P25, DiRECT, 3 months)

I know you can put it back on way faster than you take it off … [food reintroduction] made me nervous, because I felt like I tried really hard to get [the weight] off, and I didn’t want it to come straight back on.

(P29, DiRECT, 3 months)

#### 3.1.5. Overcoming Obstacles to a New Normal

At three months, the DiRECT participants identified positive shifts in their relationships with food and a heightened awareness of hunger and fullness cues following the total diet replacement phase. They described adopting more regular eating patterns and consuming smaller portions as habits they intended to maintain. Participants acknowledged the obstacles of daily life (e.g., stress and special occasions) as unavoidable, recognising weight loss and behaviour change as a work in progress rather than something that could be achieved overnight.

I don’t think I’m finished by any means, but I definitely think that I’m better than I was three months ago.

(P28, DiRECT, 3 months)

The study helped me eat less, so now I’m trying to eat less, to keep me going the healthy way.

(P42, DiRECT, 3 months)

Despite earlier concerns about ‘losing control’ around food following food reintroduction, this was rarely reported. Participants recognised the risk of reverting to old habits, but they wanted to sustain the benefits they had attained so far.

It’s just a matter of keeping it under control and not letting [my HbA1c] go back up.

(P01, DiRECT, 12 months)

Weight loss maintenance was defined by a new challenge: the pursuit of a new normal. For some, this was even more challenging than the total diet replacement phase. Participants had to negotiate a wide array of food options, select appropriate portion sizes, and, at times, manage eating-related guilt.

It was more challenging when I didn’t have the meal replacements.

(P28, DiRECT, 12 months)

Initially it was a real struggle to find, sort of, my groove of having food that was, you know, okay, and not gonna ruin all my hard work, and I became quite obsessed about it, but as it’s gone on, I’ve found things that I enjoy … so now I’ve sort of got into a routine of what I do.

(P20, DiRECT, 12 months)

Importantly, the struggles reported during the twelve-month intervention were not unique to the DiRECT participants; dietary change was described as similarly difficult in the usual care arm. Across both interventions, useful strategies included enjoying favourite foods in moderation, using smaller dinnerware, keeping a food diary, utilising meal subscription services, and staying occupied to limit mindless eating.

We got on Hello Fresh ‘cause obviously it’s portion controlled, so it minimised the overeating.

(P02, UC, 3 months)

We decided we’d get smaller plates so, you know, you feel like your plate’s full.

(P20, DiRECT, 12 months)

I have to get out and do the garden, something to take my mind away from the food and wanting it and thinking I’m hungry.

(P37, DiRECT, 12 months)

Challenges typically eased as participants were supported to adopt new strategies and establish sustainable routines, but this too required effort and adaptability.

It was hard to get rid of those bad habits.

(P33, UC, 12 months)

You’ve gotta think about what you’re eating, you can’t just eat anything, especially if you don’t wanna put the weight back on.

(P37, DiRECT, 12 months)

The cost of food was identified as a barrier to eating well and losing weight across both interventions, particularly for those with limited financial resources. ‘Healthy’ foods were perceived to be much more costly than ‘unhealthy’ alternatives, and most participants reported an increase in their usual food expenditure during this study.

We only eat what we can afford.

(P35, DiRECT, 3 months)

The healthy stuff is more expensive. It sucks.

(P20, DiRECT, 12 months)

However, working with the dietitian to identify affordable options was useful, and the ongoing benefits attained by the participants and their families typically justified any additional costs associated with consuming a healthier diet.

[The dietitian]’s shown us what is the better food at a reasonably good price.

(support person for P09, UC, 3 months)

Me and my husband agree that it’s worth it.

(P04, UC, 3 months)

#### 3.1.6. New Habits, New Perspectives

A plateau in weight loss or weight regain in the latter stages of this study was common in both interventions. Though this could be frustrating for some, others were content with their new body weight, feeling that they had achieved something they never thought possible.

After the initial weight loss it’s sort of plateaued … I’ve started putting weight back on, which I’m really gutted about.

(P20, DiRECT, 12 months)

I’ve lost the weight that I’ve been wanting to lose, and I want to lose more.

(P12, DiRECT, 12 months)

Now I find I’m not losing, but I’m more … I feel good about my body. Yeah, I feel happy … I feel comfortable about my body.

(P07, DiRECT, 12 months)

Importantly, the individuals in both interventions came to view their participation not only as a pursuit of physical change but also of personal growth. Participants differentiated their previous habits, identities, and perspectives from those of their current selves: a transformation from negative to positive.

I just went on with life thinking … I’m fat, I’m gonna stay fat, you know? I didn’t give two tosses about myself … when I actually met [the dietitian] I started feeling that I did give a toss about myself, and I needed to help myself.

(P12, DiRECT, 12 months)

Mentally and physically, it helps you as a person. You feel better in yourself … just knowing the right foods, picking the right foods. Before that, I stuck to [eating] the bad stuff … obviously it affected the way I was living. It affected my health. I mean, learning the new habits pretty much changed everything

(P31, UC, 12 months)

After twelve months, participants in both interventions felt equipped with a new knowledge of the appropriate food choices and their effects (e.g., on satiety and glycaemic control) and an improved understanding of diabetes. They had learned skills such as label reading and had adopted healthier eating habits.

I found myself going round the supermarket reading food labels … I used to laugh at people that did that.

(P08, DiRECT, 12 months)

Whereas strict compliance with the DiRECT intervention promoted success early on, food reintroduction and weight loss maintenance required greater flexibility. Participants in both interventions described shifts in their attitudes towards food, which, though challenging, reinforced the concept of long-term, sustainable lifestyle modification. Participants described choosing ‘unhealthy’ options at times, but this was no longer considered a catastrophe.

It’s just around that habit building … me and my wife, we know when a choice is not the best choice to make, but it’s also a case of not beating ourselves up for it as well because that only makes it worse.

(P06, UC, 12 months)

Saturday, Sunday is a day that we can treat ourselves to ice cream or whatever. But Monday to Friday, it’s a no-no for us.

(P07, DiRECT, 12 months)

Instead, they identified a new-found awareness of their eating habits: what, when, how much, and why they were eating, and the impacts of their choices on immediate and longer-term health outcomes.

Despite what the results say, ‘cause they are all over the show [laughing] … I think there has been an overall trend towards improvement in the way I eat and treat food.

(P06, UC, 12 months)

Oh, definitely, it’s different now [laughing]. I’d say I definitely eat different. I watch what I’m eating, and I watch how much I’m eating.

(P07, DiRECT, 12 months)

I think that I think about food differently now and actually think about what I’m putting into my body instead of just eating whatever’s there.

(P08, DiRECT, 12 months)

In contrast to the lack of confidence expressed at the beginning of this study, participants reported a greater sense of competence and self-efficacy in relation to food, and many learned to value their health more than before.

I’ve created better habits, and I’m more confident in making better choices as well, even when I go out.

(P02, UC, 12 months)

I can do this on my own now that I know what I’m doing and what it’s doing to my body and how I’m feeling, so I’m so stoked. I really am.

(P12, DiRECT, 12 months)

Potential barriers were identified, such as the many ‘unhealthy’ foods readily available from supermarkets and fast-food outlets, but, in general, participants felt equipped to manage these challenges as they arose.

There’s always gonna be days we’re home and we’re like, I really cannot be bothered cooking right now, let’s go get some fish and chips.

(P06, UC, 12 months)

I think I’m more resilient. Like I said, being in the supermarket and I’m like, no, don’t need that. Now I sort of think about, do I really need that? What will happen if I eat that?

(P08, DiRECT, 12 months)

Though many DiRECT participants felt that they would consider a total or partial diet replacement regimen to accelerate or maintain weight loss in future, this would ideally not be necessary.

I would consider using some of the meal replacement stuff … maybe one or two meals to kinda keep the calories under control sort of thing.

(P01, DiRECT, 12 months)

Rather this be the one-off, learn, tick the box, and let’s get things back in hand and not have to do that sort of dieting again.

(P43, DiRECT, 3 months)

A positive shift in mindset had occurred, and participants’ sights were set on long-term lifestyle modifications.

This study’s just allowed me to actually hone in on that long-term lifestyle change as opposed to quick fixes.

(P08, DiRECT, 12 months)

You’ve gotta make those changes in life, otherwise you’re not gonna be here that long.

(P31, UC, 12 months)

It’s gotta be a long-term, rest of your life sort of regime now.

(P43, DiRECT, 12 months)

In contrast to the temporal process of adaptation described in Theme 1, the following themes illustrate the interpersonal influences on outcomes identified by participants: the social and cultural factors relevant to their experiences at both study timepoints.

### 3.2. A Synergistic Relationship between Participant and Professional

For dietetic support to be useful, participants had to want change, be receptive to professional guidance, and be willing to initiate change in their own lives and routines. Tailored support from the study dietitian encouraged participants to value their wellbeing. Without some sense of intrinsic motivation and the active input of the participant, however, this guidance was perceived to offer little benefit: an attitude that persisted for the duration of this study. In both interventions, dietetic support and self-motivation worked together to shape participants’ overall success.

She made me understand how good I’d feel if I kinda lost the weight … She goes, ‘I don’t want you to do it for me, I want you to do it for yourself.’

(P12, DiRECT, 3 months)

Even though [the dietitian] will say this is good, this is what I need to focus on … but without me wanting to do it, and wanting to make a change, it will never happen.

(P17, UC, 12 months)

They can offer as much advice as they can, but at the end of the day, it comes down to you. Comes down to yourself, ‘cause you’re the one that’s got to do the work.

(P37, DiRECT, 12 months)

#### 3.2.1. Education and Encouragement: The Role of Dietetic Support

Before this study, participants had received weight loss advice from doctors, friends and family, colleagues, and the internet. However, this information was frequently perceived to be impractical, unsolicited, or delivered insensitively. The participants felt that doctors lacked the time and expertise to provide helpful advice regarding nutrition and physical activity.

Exercise more. Exercise more than what? Eat less. Eat less what?

(P08, DiRECT, 3 months)

They were giving me all of these other different advices that I really didn’t wanna hear.

(P12, DiRECT, 3 months)

Participants demonstrated a sense of helplessness in navigating these ‘mixed messages’ alone but wanted to avoid judgement for their body size or lifestyle choices. As a result, the participants expressed apprehension about (or even resistance to) receiving professional advice.

People telling you what is okay to eat and what is healthy to eat, that varies heaps … That’s hard going, getting all the mixed messages … Then when [the GP] talked about a dietitian … I don’t want that, thank you very much, don’t want some skinny person telling me I’m too fat.

(P20, DiRECT, 3 months)

I do struggle with my weight, so yeah, I didn’t want someone that was gonna be belittling.

(P33, UC, 3 months)

Despite their initial hesitance, the study dietitian became integral to participants’ success. The participants saw the dietitian as an essential source of professional advice, dietary education (e.g., appropriate portion sizes and healthier food substitutions), and accessible explanations regarding nutrition and health. The regularity of dietetic consults facilitated a sense of constant support and accountability, with an impending consult being a deadline for change and a means of ‘checking in’ to track progress. Furthermore, some participants reported a decline in motivation when these consults became less frequent. Even so, participants were reassured by the knowledge they could contact the dietitian for advice and encouragement outside of their scheduled visits.

She explained a lot of things to me that I didn’t really understand about my body.

(P12, DiRECT, 3 months)

That’s what’s been giving me the encouragement to do better, because I’m accountable, I guess … I go to see [the dietitian] in two weeks so I’ve at least gotta make some changes.

(P33, UC, 3 months)

It’s pretty good to see a dietitian...it helped me to stay focused.

(P42, DiRECT, 3 months)

Coming off the meal replacements, the meetings with [the dietitian] were not as frequent … You don’t have someone there every week making you accountable for your decisions … I probably didn’t think about what I was eating as much as I should.

(P01, DiRECT, 12 months)

It’s just having those regular check-ins that helped keep me on track. It just gave me a little guide on where I was at.

(P08, DiRECT, 12 months)

Participants particularly valued the dietitian’s personal and professional attributes, describing her approach as non-judgemental; tailored to individual needs, preferences, and challenges; and focused on improving wellbeing as opposed to weight loss alone.

It’s more about how you’re feeling and how you’re going, and little changes that you’ve made. So she’s real encouraging … it’s real helpful not being judged.

(P02, UC, 3 months)

[The dietitian] helped us … she really helped us. Explain how we walk through the hard time, and she explain everything … it’s really helped us … now we feel happy, and we can’t wait for the next time.

(P36, UC, 3 months)

She helped refocus me on what I was really aiming for. Not just weight loss, it was really health.

(P37, DiRECT, 3 months)

It felt like she cared about how I felt about myself, and how I wanted to feel about myself … She made me feel like a person.

(P12, DiRECT, 12 months)

At the three-month timepoint, most participants perceived continued dietetic support as crucial to their ongoing success. At twelve months, participants in both interventions expressed a preference for tailored healthy eating and weight loss advice from a nutrition specialist (i.e., a dietitian or nutritionist) as opposed to a doctor or other health professional. However, participants appreciated collaboration between the dietitian and other health professionals throughout both interventions, as this was seen to facilitate a comprehensive level of care.

I think a dietitian’s better. Yeah, obviously they’re more specialised in it. Probably I would go to a dietitian over a doctor if I wanted that.

(P33, UC, 12 months)

[Healthy eating]’s what [the dietitian]’s trained in. She has amazing knowledge of it, and she presents it beautifully, too … I mean, I’ve had lots of different dietitians over the years that have been very judgy. She’s not like that at all, which makes a huge difference.

(P20, DiRECT, 12 months)

There was always the GP back-up, so if I was concerned about stuff [the dietitian] would reach out to them for me, and then also … the pharmacist. I had discussions with her about medications I was taking.

(P20, DiRECT, 12 months)

#### 3.2.2. Determination and Discipline: The Role of Self

Alongside the role of the study dietitian was that of the individual: participants viewed themselves as equally responsible for their perceived success (or otherwise). While self-discipline was seen as a powerful driver of behaviour change and weight loss, a perceived lack of willpower could inhibit progress.

Comes down to me and the old self-discipline, motivation.

(P01, DiRECT, 3 months)

Not only was [the dietitian] helping me, but I needed to help myself to help her as well.

(P12, DiRECT, 3 months)

There is an element of personal responsibility. So it’s my responsibility to make sure I’m eating the right things.

(P25, DiRECT, 3 months)

Weight loss required intensive mental and emotional input. Some participants struggled to find and retain motivation, especially during the first three months. In contrast, existing intrinsic motivation was a key facilitator of weight loss and behaviour change for others. For example, those with a strong desire for better health could draw on such goals when faced with challenges.

It’s just a mindset of getting into a better health, so that’s the thing that kept me going.

(P07, DiRECT, 3 months)

For some participants, it was difficult to recognise achievements and retain motivation while also managing physical or mental health issues. Depression, anxiety, and other psychological issues inhibited success beyond more superficial concerns about motivation and self-control.

If my head’s messy then I eat crap food.

(P20, DiRECT, 12 months)

It’s not just about willpower or watching what you eat, it’s kind of about keeping your mind healthy too.

(P33, UC, 3 months)

### 3.3. Social Circles Shape Success

Participants’ social environments were important influences on weight loss and behaviour change, illustrated by three sub-themes: (1) support (or otherwise) from family and friends; (2) eating as a mode of connection; (3) the role of sociocultural expectations.

#### 3.3.1. Family, Friends, and Colleagues: Motivator or Burden?

Whānau (family) was a key motivator for participation, especially among those with a family history of metabolic disease who wanted to ‘stick around’ for their loved ones for as long as possible.

So that’s probably the drive behind this, knowing that my mum departed early … I wanna be here for as long as I can.

(P33, UC, 3 months)

Taking part was also seen as an opportunity to improve the health and wellbeing of whānau members—a key priority for many participants—and the experiences of each intervention were often discussed in collective terms (i.e., “we” rather than “I”).

If I do it I can help my family, and my family’s my first priority.

(P04, UC, 3 months)

We hardly eat salad at home, but now you gotta have salad when we have lunch or dinner … or some kind of vegetable. We gotta have it on the table … and we gotta have water every day, and fizzy is only treats! That’s the change we are doing and we are continuing with now … it should be there to educate not just me, but them, too.

(P07, DiRECT, 3 months)

I’m not the healthiest eater. I didn’t want that for my children.

(P27, DiRECT, 3 months)

Support from family was considered crucial in both interventions, and friends and colleagues often provided additional encouragement. Reciprocally, participants felt they could support their loved ones to make positive changes as well.

My husband, my family, my kids, working together to achieve it.

(P04, UC, 3 months)

You do need the support of your family.

(P35, DiRECT, 3 months)

My good friend and I … she had a gastric bypass. She must be nearly three years out, so she made the changes before me, but it’s been really good. We feed off each other.

(P20, DiRECT, 12 months)

Though the DiRECT participants valued the social support they received, some felt the total diet replacement phase was (or would be) easier without having others to care for at the same time.

Living by myself makes things a lot easier.

(P01, DiRECT, 3 months)

I think it would have made it easier if it was just on my own with no kids, family … to prepare food for and everything.

(P07, DiRECT, 3 months)

Nevertheless, family life could be challenging for participants in both interventions. Difficulties emerged when others’ eating habits failed to align with participants’ intentions. Some participants found it hard to meet whānau members’ needs and preferences while still achieving their own dietary goals.

With me eating differently it’s been quite tricky … my daughter hates it because she’s a meat and potato lover … my biggest obstacle was my husband, because he doesn’t like rabbit food.

(P20, DiRECT, 3 months)

It’s just when you are a family and you’re trying to feed kids … no matter how hard you try, they are not as keen with vegetables and salads, so trying to do a family meal that is well balanced … it’s hard.

(P28, DiRECT, 12 months)

In contrast, participants felt most successful when others in the household were also receptive to dietary change. For example, one participant introduced the Māori concept of a shared kaupapa, referring to her family’s work towards a common goal or purpose.

It’s always helpful when everyone in your house is on the same kaupapa.

(P02, UC, 3 months)

Total diet replacement was sometimes perceived by others as too extreme or unsafe, and navigating these attitudes was an additional challenge for DiRECT participants. While discussion with others could be a valuable source of support or motivation, it could also make for uncomfortable conversations, unrealistic expectations, and even conflict.

Everyone sort of freaked out, and then they sort of held me accountable as well.

(P08, DiRECT, 3 months)

I sort of grew up with parents that were very big on … you need to eat because if you don’t, you’re not gonna be well … my mum lives with me, so she was not very happy at all about the whole process.

(P20, DiRECT, 3 months)

Their opinion was that they think it’s too restrictive and there’s no way they could’ve done it. It almost motivated me more to be fair, to show them that it can be done.

(P28, DiRECT, 3 months)

#### 3.3.2. Food as a Facilitator of Connection

In both interventions, food facilitated interaction and connection in participants’ day-to-day lives (e.g., eating with family or coffee with colleagues) and on special occasions (e.g., Christmas or birthdays). The social nature of eating could be particularly difficult for the DiRECT participants during the three months of total diet replacement. For example, participants described preparing home-cooked meals for other family members then watching as these were consumed as mentally testing. Eating separately to the rest of the household, taking diet replacements to restaurants, or avoiding social activities altogether helped to mitigate these issues, but these strategies could also lead to feelings of isolation.

If they were to eat their food they would go away and would leave me … I know they were trying to help, but in a way, I felt like I was abandoned.

(P07, DiRECT, 3 months)

You look like the weirdo with your shake in the corner.

(P20, DiRECT, 3 months)

It was easy to just stick by myself … not mingle with anyone at cafés and that.

(P37, DiRECT, 3 months)

At three months, the DiRECT participants saw a return to social eating as a key advantage of food reintroduction. Even so, they felt the positive outcomes attained during the total diet replacement phase were worth the social sacrifices required.

The positives have way outweighed that negative of not being quite as social in the beginning.

(P25, DiRECT, 3 months)

#### 3.3.3. Navigating Norms and Breaking (Sociocultural) Barriers

Social and cultural norms and expectations surrounding food had shaped participants’ lifelong eating behaviours, sometimes in a negative way.

I was eating to please everybody else and not eating for myself.

(P12, DiRECT, 12 months)

We were taught, you get a plate of food, you eat the lot, you know what I mean? And that was my habit. Eat the lot and doesn’t matter how I feel … I’ve actually changed in the last three months. I don’t have to eat all that food.

(P33, UC, 3 months)

Participants’ perceptions of food and eating were closely linked to their identities and values. Food was central to cultural activities and family gatherings and was an important means of giving and receiving hospitality, particularly for those from Māori or Pacific Island backgrounds.

We’re Islanders. We like family gathering, and we love food.

(P42, DiRECT, 3 months)

While many participants enjoyed traditional foods and ways of eating, these were often perceived as ‘unhealthy’ or incompatible with weight loss. Alongside the ever-present challenge of hedonic temptation, denying an offer of food or wasting food conflicted with personal and cultural values. As such, the cultural significance of food remained an ongoing challenge for many participants.

People will come and then they like cook all this food that I can’t necessarily eat, but it kills me to waste food, so it was a lot of conflict, I suppose, when people visit.

(P02, UC, 3 months)

Being an Islander we were never told to eat healthy.

(P04, UC, 3 months)

The hardest part’s gonna be with all the events that you go to. Birthdays, farewells … especially as Māori, ‘cause any event, good thing, bad thing, there’s always food. Yeah, there’s always that little thing in the back of my head that dad used to tell me: ‘When someone offers you something, don’t say no.’

(P08, DiRECT, 3 months)

Overcoming these norms and expectations was challenging yet necessary for success. Participant-led solutions were important, not only for the benefit of the individual but also for that of their loved ones.

So it’s just learning to, if I do go somewhere … an event where there’s food like that, trying to rush and just dish up my own plate before someone does it for me. Just little things like that I guess will be the hardest part.

(P08, DiRECT, 3 months)

I was taught bad habits when I was young … so I’ve had to make more changes in my life, and pass that on to my grandkids.

(P33, UC, 3 months)

## 4. Discussion

We have considered the acceptability of the DiRECT intervention and explored participant experiences of both DiRECT and dietitian-supported usual care in a predominantly Māori and Pacific Island population living with type 2 diabetes or prediabetes in Aotearoa New Zealand.

Participants recounted a history of unsupported and unsuccessful attempts at weight loss prior to enrolment in this study, contributing to an initial sense of helplessness and hesitance towards accepting professional advice. Despite this, ongoing dietetic support bolstered participants’ confidence and resilience in the subsequent twelve months. Both DiRECT and dietitian-supported usual care required persistent effort to facilitate physical, psychological, and behavioural change, instigating positive transformations among participants. For the DiRECT participants, each phase of the intervention (total diet replacement, food reintroduction, and supported weight loss maintenance) posed unique challenges, but these could be overcome with input from both the participant and the study dietitian. Physical hunger and psychological challenges were apparent during the three-month total diet replacement phase, but rapid weight loss and a sense of control provided the motivation to continue. Despite the perceived benefits of the food reintroduction phase (greater choice, flexibility, and opportunities to socialise), many participants feared that they would regress to former habits and regain any weight loss achieved. Indeed, the longer weight loss maintenance phase required effort (deciding what, when, and how much to eat), but participants drew strength from their growing confidence and the knowledge that the dietitian remained present to support them in adopting and sustaining a ‘new normal.’ As such, the DiRECT intervention was considered a rewarding and acceptable experience. Within both interventions, interactions between the participants and their family, friends, and wider communities were identified as key influences on success, but also potential barriers. 

Previous research has found DiRECT to be an acceptable approach for weight loss among people with type 2 diabetes [14,15,16]. In line with the process of adaptation we have reported, Rehackova et al. describe a “fluctuation of experience” within the original DiRECT-UK trial [14] (p. 4). The participants in our study and the DiRECT-UK trial each identified new and reoccurring challenges requiring resilience and tailored support. In fact, many of the experiences described by our participants appear universal: a desire for weight loss and better health as a stimulus for participation in a total diet replacement intervention [14,15,16,46,47]; the abrupt but worthwhile adjustment to a total diet replacement regimen [14,15,16,46]; professional support as a source of accountability [14,15,48]; drawing on rapid and early weight loss as an important motivator [14,15,48]; navigating the “disruption to adaptation” [14] (p. 7) posed by food reintroduction; the challenging realities of weight loss maintenance [14,16,46,47,48,49]; and the development of “behavioural autonomy” [49] (p. 956). As we identified, the timing and intensity of the challenges faced by individuals vary, and additional personal barriers frequently emerge [14,15,46,47,48]. 

Several of the perspectives and experiences we have reported were not unique to the DiRECT intervention, particularly those relevant to interpersonal interactions and influences. Rather, these were shared by both DiRECT and dietitian-supported usual care participants. Importantly, these findings have been highlighted in previous qualitative studies reporting the experiences of people with type 2 diabetes and prediabetes within real-world care [50,51,52] and in various lifestyle interventions [53,54,55,56,57]. People with type 2 diabetes often desire empathetic, person-centred support to improve their health [50,51] and express concerns about motivation and accountability when professional guidance is reduced or ceased [53,54]. Sociocultural factors may both facilitate and impede lifestyle modification efforts, irrespective of intervention type or social context. For example, social obligations and expectations challenge adherence to time-restricted eating [55] and more conventional dietary interventions [56]. As we also observed, family members have been recognised as both positive and negative influences on eating and activity behaviours [52,56,57]: providing social support or “leading each other astray” [52] (p. 190).

Our research contributes a unique set of perspectives regarding DiRECT and dietitian-supported usual care to the existing literature, reflecting the experiences of participants engaged in cultures where food and eating carries increased sociocultural significance. In contrast, previous qualitative evaluations of DiRECT have been conducted in samples with limited ethnic and cultural diversity [14,16]. 

Within te ao Māori (the Māori worldview), food is intrinsic to every aspect of health and wellbeing. Similarly, food both sustains and gives meaning to life in many Pacific populations. Within each of these cultures, the giving, receiving, and sharing of food reflects important beliefs and values such as showing manaakitaka/manaakitanga (hospitality, generosity, and respect) and reciprocity [26,58,59]. It is often through food that relationships are “forged, maintained and strengthened” [26] (p. 8) and cultural knowledge is transmitted between generations. As such, food is understood not only as a source of sustenance but also as a central facilitator of connection with one’s family, their wider community and culture, and their environment in both Māori and Pacific cultures. An important finding of this present study is, therefore, the central role played by these social and cultural factors in shaping our participants’ perceptions and experiences of both DiRECT and dietitian-supported usual care. 

Social gatherings involving food have been identified as potential barriers to adherence in previous interventions incorporating total diet replacement [16,48]. For our participants, however, such challenges were intensified by an underlying cultural code surrounding food: eating differently in an attempt to lose weight often meant breaking unspoken social rules [26,60] irrespective of the assigned intervention. In another New Zealand study, Māori and Pacific people with prediabetes similarly identified cultural expectations for reciprocity (giving and receiving food) as a key barrier to improved nutrition [56]. Therefore, the same social and cultural norms, values, and expectations that uphold cultural identity may simultaneously hamper individuals’ dietary modification efforts [26,52,56,61,62]. Within our study population, we have identified the high sociocultural importance of food and shared eating, the barriers to weight loss this may create, and the overall acceptability of DiRECT, including a willingness to use meal replacements for future weight loss or weight loss maintenance. Given these findings, it might be reasonable to expect a partial meal replacement intervention to be an even more acceptable approach in some circumstances. Although the acceptability of partial meal replacement was not explicitly explored with our participants, such a strategy might better uphold social and cultural values, promote greater adherence, and facilitate improved long-term weight and health outcomes for some individuals, provided slower weight loss is not of concern.

Despite both interventions in this present study targeting individual weight loss, our participants positioned their involvement in this study within a broader social context [52]. Though often translated as ‘family,’ the Māori term ‘whānau’ encompasses a complex and dynamic understanding of physical, spiritual, and emotional relationships [63]. Whānau played a far greater role than merely providing side-line support; maximising time with loved ones and setting a positive example for future generations were key reasons to participate. Importantly, the expectations and experiences of each intervention were discussed not only in relation to the individual but also to the people around them. As such, our participants reiterated the fundamental principles of community, collectivism, and family common to the worldview of both Māori and Pacific peoples [64,65] and that of many indigenous cultures [66].

In both interventions, participants described building a strong professional relationship with the study dietitian in which they felt accepted and listened to; the “compassionate, empathetic, and responsive” [67] (p. 351) nature of the support received was a key determinant of their perceived success. In a kaupapa Māori context, this reflects values of whanaukataka/whanaungatanga or honoka/hononga (interconnectedness) and whakawhanaukataka/whakawhanaungatanga (relationship building) [68]. These principles are crucial when working with Māori and Pacific communities: positive health outcomes require reciprocal and trusting relationships between culturally competent healthcare professionals and their patients [68,69,70]. Culturally tailored support for weight loss and weight loss maintenance should acknowledge the social and cultural strengths and challenges important to the individual, such as the centrality of food to cultural identity, the norms and expectations surrounding eating, and the importance of family and wider community identified by our participants. Our study comes with several strengths, as well as some limitations. In conducting a reflexive analysis we acknowledge that, despite the multicultural makeup of our research team, our views as researchers may differ from those of our participants. We also acknowledge the heterogeneity in perspectives within and between Māori and Pacific cultures; this analysis captures only the essence of their similarities [71]. Our analysis is reflective of our participants’ experiences of the weight loss interventions at the time and cannot be directly generalised to other sociocultural contexts. Any application of our research must appreciate the diversity of perspective and experience both across the different population groups and between the individuals within those groups. All the participants worked with the same dietitian within their primary care provider for the full twelve-month duration of the interventions. This contributed to participants’ positive experiences of each intervention, but such consistency may not be feasible in all settings due to factors including limited funding and staff availability. All participants were invited to be interviewed at both time points, irrespective of their quantitative results (e.g., weight loss), allowing us to capture a broad range of perspectives. It is possible that participants who did not contribute to the analysis may have had differing experiences from those we have described. Almost all the interviews were conducted by the same researcher at three and twelve months, enhancing rapport with the participants and continuity between each timepoints. To our knowledge, this is the first qualitative evaluation of a weight loss intervention incorporating total diet replacement conducted with participants from cultures with strong emphases on food and eating. Unlike previous qualitative evaluations of DiRECT conducted in predominantly European populations [14,15,16], we included participants with any duration of type 2 diabetes, as well as those with prediabetes. Our findings therefore reflect perspectives across a broad spectrum of impaired glucose control. Further, this is the first of these studies to examine participants’ experiences of usual care alongside those of DiRECT. This enabled us to identify the perspectives and experiences shared by both groups of participants and comment more specifically on the acceptability of the DiRECT intervention. 

## 5. Conclusions

Participants’ xperiences of the DiRECT intervention were marked by numerous challenges, but these were overcome when dietetic support was paired with effort and adaptability. Early weight loss and a sense of routine promoted motivation and adherence during the total diet replacement phase, despite its physical and psychological challenges. Next, the perceived risks of food reintroduction were balanced by the benefits of greater food flexibility. Finally, the effort of adopting and sustaining new habits throughout weight loss maintenance was supported by the participants’ growing sense of confidence and self-efficacy. Therefore, the DiRECT intervention was considered an acceptable approach to weight loss and weight loss maintenance in this population of adults with type 2 diabetes or prediabetes and strong cultural emphases on food and shared eating. Across both interventions, social and cultural factors were recognised as key determinants of success or failure, and tailored support from an empathetic health professional was considered essential in improving all aspects of the participants’ health and wellbeing. Our findings reiterate the importance of individualised and culturally relevant behavioural support for effective weight loss and weight loss maintenance. These observations will be valuable in informing future adaptations of DiRECT and potentially many other lifestyle modification interventions among socially and culturally diverse population groups.

## Figures and Tables

**Table 1:** Participants’ characteristics and included interviews.

Intervention	Participant	Sex	Diagnosis	Interview Included	
				3 Months	12 Months
DiRECT	P01	M	T2D	✓	✓
	P07	F	T2D	✓	✓
	P08	M	T2D	✓	✓
	P12	F	T2D	✓	✓
	P20	F	Pre	✓	✓
	P25	F	T2D	✓	–
	P27	F	Pre	✓	–
	P28	F	T2D	✓	✓
	P29	F	Pre	✓	–
	P35	F	Pre	✓	✓
	P37	F	T2D	✓	✓
	P41	M	T2D	✓	–
	P42	F	T2D	✓	–
	P43	M	T2D	✓	✓
Dietitian-supported usual care	P02	F	T2D	✓	✓
	P04	F	T2D	✓	–
	P06	M	T2D	–	✓
	P09	F	T2D	✓	✓
	P17	F	T2D	✓	✓
	P19	F	Pre	✓	–
	P24	M	Pre	✓	–
	P26	F	Pre	✓	–
	P30	F	T2D	✓	–
	P31	M	Pre	✓	✓
	P33	F	T2D	✓	✓
	P36	F	T2D	✓	–

F: female; M: male; T2D: type 2 diabetes; Pre: prediabetes; and ✓: included.

## Data Availability

Deidentified data may be made available upon reasonable request to the corresponding author due to privacy, ethical, and legal reasons.

## Supplementary Materials

The following supporting information can be downloaded at https://www.mdpi.com/article/10.3390/nu16121853/s1: completed COREQ checklist and the semi-structured interview guides used at 3 and 12 months.

## Glossary

Honoka/hononga	bond, connection, or relationship
Kaupapa	purpose or initiative informed by principles, values, and ideas
Kaupapa Māori	Māori customary practice
Manaakitaka/manaakitanga	act of showing hospitality, care, generosity, and respect
Māori	the Indigenous people of Aotearoa New Zealand
Te ao Māori	the Māori world(view)
Whakawhanaukataka/ whakawhanaungatanga	act of establishing relationships
Whānau	family or extended family
Whanaukataka/whanaungatanga	sense of kinship or connection
