# Supplementary material for: Experiences and Acceptability of a Weight Loss Intervention for Diabetes (Diabetes Remission Clinical Trial—DiRECT) in Aotearoa New Zealand: A Qualitative Study within a Pilot Randomised Controlled Trial
Source: Nutrients. 2024 Jun 13;16(12):1853. doi: 10.3390/nu16121853 (PMC11206426; doi:10.3390/nu16121853)
Supplement: Supplementary file 1 [file nutrients-16-01853-s001.zip › Supplementary file 1. COREQ checklist.pdf]

Consolidated criteria for reporting qualitative studies (COREQ): 32-item checklist.

Manuscript: Experiences and acceptability of a weight loss intervention for diabetes (DiRECT) in Aotearoa New Zealand: a qualitative study within a pilot randomised controlled trial

**No. Item**	**Guide questions/description**	**Reported on Page #**
**Domain 1: Research team and reflexivity**		
Personal Characteristics		
1. Interviewer/facilitator	Which author/s conducted the interview or focus group?	p. 3-4 (section 2.4.)
2. Credentials	What were the researcher's credentials? E.g. PhD, MD	p. 4 (section 2.4.)
3. Occupation	What was their occupation at the time of the study?	p. 4 (section 2.4.)
4. Gender	Was the researcher male or female?	p. 4 (section 2.4.)
5. Experience and training	What experience or training did the researcher have?	p. 4 (section 2.4.)
Relationship with participants		
6. Relationship established	Was a relationship established prior to study commencement?	p. 4 (section 2.4.)
7. Participant knowledge of the interviewer	What did the participants know about the researcher? e.g. personal goals, reasons for doing the research	p. 4 (section 2.4.)
8. Interviewer characteristics	What characteristics were reported about the interviewer/facilitator? e.g. Bias, assumptions, reasons and interests in the research topic	p. 4 (section 2.4.)
**Domain 2: study design**		
Theoretical framework		
9. Methodological orientation and Theory	What methodological orientation was stated to underpin the study? e.g. grounded theory, discourse analysis, ethnography, phenomenology, content analysis	p. 4-5 (section 2.5.)
Participant selection		
10. Sampling	How were participants selected? e.g. purposive, convenience, consecutive, snowball	p. 5 (participants enrolled in RCT)
11. Method of approach	How were participants approached? e.g. face-to-face, telephone, mail, email	p. 5 (section 2.6.)
12. Sample size	How many participants were in the study?	p. 5 (section 2.6.) and Figure 1
13. Non-participation	How many people refused to participate or dropped out? Reasons?	Figure 1
Setting		
14. Setting of data collection	Where was the data collected? e.g. home, clinic, workplace	p. 3 (via telephone) (section 2.4.)
15. Presence of non-participants	Was anyone else present besides the participants and researchers?	p. 4 (section 2.4.)

16. Description of sample	What are the important characteristics of the sample? e.g. demographic data	p. 5 (section 3.) and Table 1
Data collection		
17. Interview guide	Were questions, prompts, guides provided by the authors? Was it pilot tested?	p. 3 (section 2.4.) and supplementary material
18. Repeat interviews	Were repeat interviews carried out? If yes, how many?	p. 2 (interviews at 3 and 12 months) (section 2.1.)
19. Audio/visual recording	Did the research use audio or visual recording to collect the data?	p. 4 (section 2.4.)
20. Field notes	Were field notes made during and/or after the interview or focus group?	p. 4 (section 2.4.)
21. Duration	What was the duration of the interviews or focus group?	p. 4 (section 2.4.)
22. Data saturation	Was data saturation discussed?	p. 5 (section 2.5.)
23. Transcripts returned	Were transcripts returned to participants for comment and/or correction?	p. 4 (section 2.5.)
**Domain 3: analysis and findings**		
Data analysis		
24. Number of data coders	How many data coders coded the data?	p. 4 (section 2.5.)
25. Description of the coding tree	Did authors provide a description of the coding tree?	p. 4 (section 2.5.)
26. Derivation of themes	Were themes identified in advance or derived from the data?	p. 4 (section 2.5.)
27. Software	What software, if applicable, was used to manage the data?	p. 4 (section 2.5.)
28. Participant checking	Did participants provide feedback on the findings?	p. 4 (section 2.5.)
Reporting		
29. Quotations presented	Were participant quotations presented to illustrate the themes/findings? Was each quotation identified? e.g. participant number	pp. 8-18 (section 3.)
30. Data and findings consistent	Was there consistency between the data presented and the findings?	pp. 7-18 (yes, as illustrated in section 3.)
31. Clarity of major themes	Were major themes clearly presented in the findings?	pp. 7-18 (section 3.) and Figure 2
32. Clarity of minor themes	Is there a description of diverse cases or discussion of minor themes?	pp. 7-18 (section 3.) and Figure 2

Adapted from: Tong A., Sainsbury P., Craig J. Consolidated criteria for reporting qualitative research (COREQ): a 32-item checklist for interviews and focus groups. *Int J Qual Health Care* **2007**, *19*, 349-57. doi:10.1093/intqhc/mzm042
