# Supplementary material for: Experiences and Acceptability of a Weight Loss Intervention for Diabetes (Diabetes Remission Clinical Trial—DiRECT) in Aotearoa New Zealand: A Qualitative Study within a Pilot Randomised Controlled Trial
Source: Nutrients. 2024 Jun 13;16(12):1853. doi: 10.3390/nu16121853 (PMC11206426; doi:10.3390/nu16121853)
Supplement: Supplementary file 1 [file nutrients-16-01853-s001.zip › Supplementary file 2. Interview guides.pdf]

Semi-structured interview guide for participants at 3 months

Introduction: *Introduce self/role in study.* You've just completed the first 12 weeks or 3 months of the study, so I'd like to ask you some questions about your experience during that time. *Check if participant has any questions before continuing.*

Participant experiences with the study

The purpose of the study is to help people lose weight and keep weight off.

1. How did you go over the past 12 weeks?
2. What were the main things that helped you over the past 12 weeks?
3. If there were issues over the past 12 weeks, what were they?
4. What would have made weight loss easier for you over the past 12 weeks?
5. What motivated you to join the study?
6. Did this motivation change during the first 12 weeks?

Participant translation into behaviour change

We asked you to change what and how much you ate during this first part of the study.

7. How did that go for you?
8. What was it like changing to new foods?
9. What was it like eating less?
10. How different has this been to what you ate before joining this study?
11. How did it feel changing from what you did eat to eating during the study?
12. What benefits, if any, have you noticed with this change in what and how much you ate?

Enablers and barriers to adherence

13. Can you tell me about any support you received during the past 12 weeks?
14. Were the foods you were asked to eat easy to prepare?
15. As a result of being in this study, did your family/whānau/friends change the way they ate as well?
16. (If yes, what benefits did you see as a whānau? If not, why do you think this was?)
17. Did other things affect how you went during the past 12 weeks?

Potential prompts, if needed:

a. *Children*
b. *Family*
c. *Social activities*
d. *Time*
e. *Busy lifestyle*
f. *Peer pressure*
g. *Seeing benefits*

Past experience and role of health professionals in giving support

People wishing to lose weight are often given a lot of healthy lifestyle advice. Before joining this study,

18. Can you tell me what advice you'd been given to lose weight?
19. Who gave you this advice?
20. In general, where do you get information about healthy eating?
21. Should GPs provide healthy eating advice?
22. Before this study, did your GP provide healthy eating advice?
23. Before this study, what support did you want to help lose weight?

Future expectations

Now that you've finished the first 12 weeks

19. What do you think will happen with your food intake going forwards?
20. Have you learnt anything in the past 12 weeks that might help you going forwards?
21. How confident are you that you can continue working on weight loss?
22. Can you think of anything more that might help you?
23. Are there any future barriers to weight loss that might come up for you?

******Intervention group only – resuming solid food intake******

24. How do you feel about returning to eating solid foods?
25. Are there benefits with returning to solid foods?
26. Are there risks with returning to solid foods?
27. Thinking forward, would you go back to full meal replacement again if you needed to?

Conclusion: *Ask if participant has any further comments about time on study. Thank participant for giving up time and sharing their feedback and experiences.*

Semi-structured interview guide for participants at 12 months

Introduction: *Re-introduce self/role in study.* Last time we spoke you'd completed the first 3 months of the study and shared a bit about your experience at that point. Now that you've been part of the study for 12 months, I'd like to ask you some questions about your experience in the last 9 months, and overall. *Check if participant has any questions before continuing.*

Participant experiences with the study

1. What did you think about being a part of this 12-month trial?
2. More specifically, how did you go in the past 9 months?
3. What do you think about your body weight over the last 12 months?
4. What do you think about your diabetes control over the last 12 months?
5. What support was available to you as part of this study? I.e. What did Te Kāika offer?
6. What support did you use as part of this study?
7. Was there more Te Kāika could have done to support you?

Enablers and barriers to adherence

8. Were there things that helped you in the last year?
9. Were there any issues or challenges that made the last year difficult for you?

Participant benefits with trial participation

10. Have you noticed any benefits from making these changes?
11. Have any of your whānau/family or friends made changes too, as a result of you being in the study?

Participant translation into behaviour change

Throughout the study you've been asked to make changes to what you eat, and how much you eat.

12. Was it easy for you to find and buy the foods recommended to you during this study?
13. Were the recommended foods easy to prepare?
14. Did you like eating any of these new recommended foods? If yes- how did you eat them.
15. What has it been like eating different amounts of food over the past year?
16. Do you think you eat differently now than before the study?

******Intervention group only – resuming solid food intake******

A big part of the last nine months was the move back to eating meals.

17. How did that go for you?
18. What was the best part about going back to real foods?
19. What was the hardest part about going back to real foods?
20. Did you go back to the meal replacement sachets at all in the last 9 months? If yes, how was that?
21. Would you use these meal replacements again in the future if you needed to? Short term/long term/some meals/ all meals etc?

Past experiences and role of health professionals in giving support

22. Thinking about any weight loss attempts before this study, was the last year better/worse/same?
23. If you were looking for information about healthy eating, where would you go now?
24. Do you think that your local doctor should give you healthy eating advice?
25. Do you think that your local doctor should give advice to lose weight?
26. What sort of professions would you go to for healthy eating advice or advice to lose weight?

Future expectations

27. Now that you've finished the study, what happens with your food choices going forward?
28. Have you learned anything useful that will help you continue on your own?
29. Are you confident about maintaining any weight loss?
30. Are you confident about maintaining any changes to what you eat?

Conclusion: *Ask if participant has any further comments about time on study. Thank participant for giving up time and sharing their feedback and experiences.*
